# Coexistence of brain capillary telangiectasia and venous angioma: A case report and literature review

**DOI:** 10.1002/ccr3.8819

**Published:** 2024-05-10

**Authors:** Moaz O. Moursi, Anas Alsadi, Yousra Ali, Jouhar Kolleri, Tanweer Hussein

**Affiliations:** ^1^ Department of Internal Medicine Hamad General Hospital Doha Qatar; ^2^ College of Medicine, QU Health Qatar University Doha Qatar; ^3^ Department of Radiology Hamad General Hospital Doha Qatar

**Keywords:** capillary telangiectasia, case report, intracranial bleeding, vascular malformations, venous angioma

## Abstract

While Cerebral vascular malformations exhibit distinct clinical and radiographical features, rare instances of coexisting lesions occur. This case report sheds light on the rare coexistence of brain capillary telangiectasia and venous angioma in a patient presenting with a seizure attributed to frontal lobe bleeding. Though often silent, brain capillary telangiectasia can manifest with serious life‐threatening intracranial bleeding. Therefore, in cases of spontaneous intracranial bleeding, an MRI of the head is crucial to rule out such cerebral vascular malformations.

## 1 INTRODUCTION

Brain capillary telangiectasia (BCT) is a small area of abnormally dilated capillary network in otherwise normal brain parenchyma. Although BCT is most commonly located in the pons,^1^, ^2^ they have been described throughout the brain.^3^ The prevalence of BCT, based on autopsy and magnetic resonance imaging (MRI) reports, has been estimated to be between 0.4% and 0.7%.^2^, ^4^

Cerebral vascular malformations, which include arteriovenous malformations, cavernous malformations, capillary telangiectasia and venous angiomas, each exhibit distinct clinical and radiographical features.^1^, ^5^ However, it is possible for multiple lesions to coexist in the same patient.^5^ In this case, we present a rare association between capillary telangiectasia and venous angioma.

## 2 CASE HISTORY/EXAMINATION

A 31‐year‐old previously healthy man was brought to emergency room after a witnessed episode of generalized tonic clonic convulsion. The episode had lasted for several minutes and aborted spontaneously. Upon the arrival of the paramedics, the patient was already back to his baseline with stable vital signs.

He reported 2 days history of severe, progressive frontal headache associated with nausea, and repeated vomiting. He denied any weakness, numbness, change in vision, photophobia, phonophobia, dizziness, fever, joint pain, skin rash, or recurrent ulcers.

There had been no recent history of trauma, falls, or long‐distance travel. The patient had no significant past medical or surgical history, and he was not taking any medication or over‐the‐counter treatment. Family history was unremarkable. He is a nonsmoker with no history of alcohol or drug use.

On physical examination, he was conscious and oriented, with a Glasgow coma scale of 15. There were no signs of meningeal irritation, and there were no focal neurological deficits. Cranial nerves, motor function, sensory function, and cerebellar examination were all normal. The rest of the systemic examination did not reveal any remarkable findings, and the patient's vital signs remained stable from admission until discharge.

## 3 METHODS (INVESTIGATIONS, DIFFERENTIAL DIAGNOSIS, AND TREATMENT)

Blood work, including a complete blood count, electrolytes, renal function, liver function, coagulation profile, inflammatory markers, viral hepatitis, human immunodeficiency virus, and HbA1c, all came back within normal limits.

**FIGURE 1:**
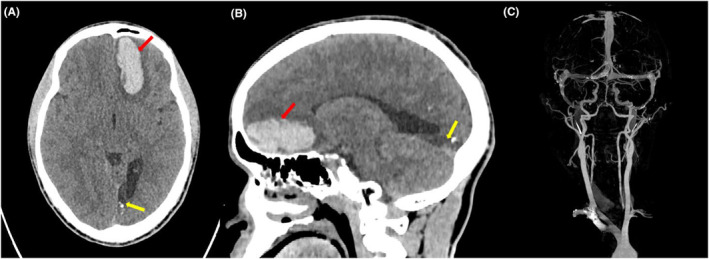
Plain CT head (A) Axial, and (B) Sagittal cuts showing an intra‐axial hematoma in the left frontal region (red arrows) with surrounding perifocal edema. A hypodense area in the left occipital region (yellow arrows) with calcifications and ex vacuo dilatation of the posterior horn of the left lateral ventricle due to the previous ischemic insult was also seen. (C) A CT intracranial angiogram is unremarkable.

An unenhanced computed tomography (CT) scan of the head revealed an intra‐axial hematoma in the left frontal region measuring 5 × 2 × 2 cm, along with surrounding edema. There was also a hypodense area in the left occipital region with calcifications and ex vacuo dilatations of the posterior horn of the left lateral ventricle, likely due to a previous ischemic insult (Figure 1A, B).

A CT intracranial angiogram demonstrated a normal course and caliber of bilateral common carotid, internal carotid, anterior and middle cerebral arteries, with good contrast enhancement. Bilateral vertebral, basilar, and posterior cerebral arteries were also patent. No evidence of arterial occlusion, aneurysm, or vascular malformation were noted (Figure 1C).

**FIGURE 2:**
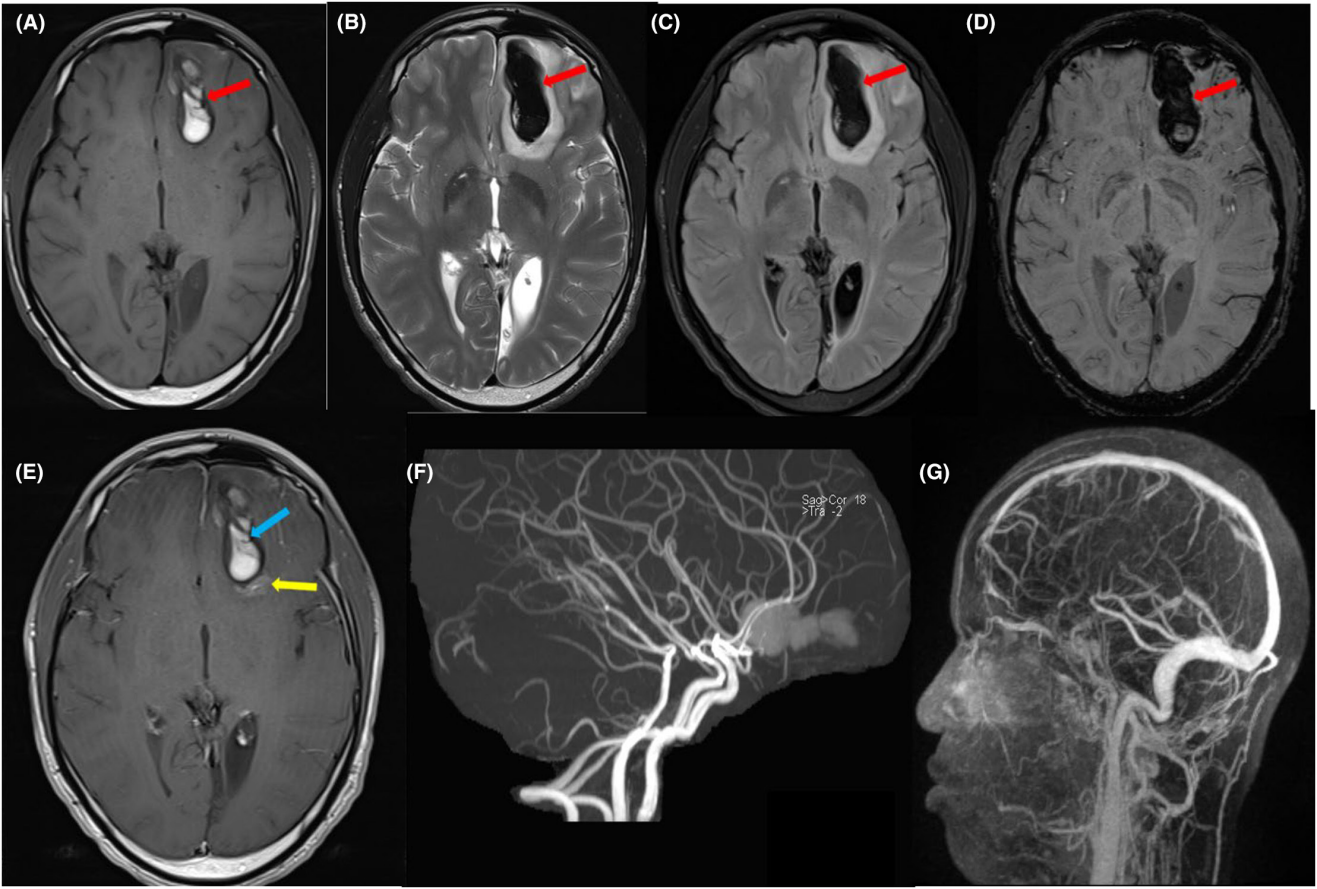
MRI head with contrast (A) T1, (B) T2, (C) FLAIR, (D) SWI, and (E) T1 post contrast shows the left frontal parasagittal hematoma with high signal intensity on T1, low signal in T2 and FLAIR, and significant SWI blooming (red arrows). Mild surrounding edema is causing a mass effect. There is no pathological post‐contrast enhancement suggesting tumor (blue arrow); however, there is a contrast blush posterior to the hematoma (yellow arrow). (F) Intracranial MRA is unremarkable with no major vascular occlusion, stenosis, aneurysm, or AVM. (G) MRV appears unremarkable with no filling defects.

To further investigate the possibility of cavernoma, head MRI with contrast was performed. The results showed a left frontal parasagittal parenchymal hematoma with low T2/FLAIR signal intensity, high T1 signal intensity (early subacute) and significant SWI blooming. There was mild surrounding edema causing a mass effect in the form of effacement of the related brain sulci and effacement of the frontal horn and anterior part of the body of the left lateral ventricle. There was no definite pathological postcontrast enhancement suggesting a tumor; however, there was an 18 mm contrast blush just posterior of the hematoma. This lesion was suggestive of capillary telangiectasia with a central venous angioma. Left occipital cortical subcortical areas of encephalomalacia, gliosis, volume loss, and underlying hemosiderin deposition, along with ex vacuo dilatation of the occipital horn and trigone of the left lateral ventricle, were seen, mostly related to a previous insult. Intracranial arterial magnetic resonance angiography (MRA) and Magnetic resonance venography (MRV) appeared unremarkable (Figure 2).

After collaborative discussions involving the neurology, neurosurgery and radiology teams, we collectively reached a consensus that the patient's seizure can be attributed to the left frontal hematoma, which itself was caused by the capillary telangiectasia alongside with the coexisting central venous angioma. Subsequently, a conservative approach was adopted, and the patient was kept on levetiracetam 500 mg twice daily. He was also scheduled for outpatient MRI/MRA follow‐up after 8 weeks. At the time of discharge, the patient's clinical status remained stable, and he was doing well with no changes observed.

## 4 CONCLUSION AND RESULTS (OUTCOME AND FOLLOW‐UP)

At the 2‐month follow‐up appointment, the patient was doing well with no seizure recurrence. He confirmed full compliance with the treatment and reported no side effects. Repeated MRI/MRA showed a stable left frontal haematoma with encephalomalacia and gliosis, as well as a stable left occipital parasagittal old insult.

## 5 DISCUSSION

While BCT often remains asymptomatic, there have been reported instances of variable neurological symptoms associated with the condition. These symptoms include bleeding, headache, seizure, weakness, vertigo, and paraesthesia.^6^ Across two population‐based case series, 8 out of 132 patients (6%) had symptoms.^7^, ^8^ In a single review of 99 patients with symptomatic BCT, symptomatic hemorrhage accounted for almost one‐third of cases, with the frontal lobe being the most common supratentorial bleeding site. The remaining two‐thirds of cases presented with headaches and various neurologic signs such as visual disturbances, dizziness, and seizures.^1^

BCT is thought to be congenital, resulting from a failure in the involution of brain capillaries during the first trimester of development.^9^ However, the rare incidence of BCT among the pediatric population may suggest an acquired pathophysiology.^10^ In a study by Ishigami et al., 43 selected genes were analyzed in a patient with a vascular anomaly and BCT with a positive family history of vascular malformation; none of them revealed any pathogenic variant.^11^ Somatic mutations are difficult to detect in BCT patients due to their inaccessibility within the brain parenchyma.^11^

BCT is classified as one of the four major types of cerebral vascular malformations, which also include arteriovenous malformations, cavernous malformations, and venous angiomas.^12^ While BCT is usually observed as a solitary finding, it may be associated with hereditary conditions such as hereditary hemorrhagic telangiectasia (Osler‐Weber‐Rendu syndrome) and Sturge–Weber syndrome.^8^ Furthermore, BCT may coexist with other venous anomalies, including venous angiomas.^13^

The association between BCT and venous anomalies suggests a potential shared underlying pathology.^14^ McCormick et al were the first to describe this association in 1993.^15^ Subsequent reports have indicated that the coexistence of BCT and venous anomalies can be found in up to 20% of symptomatic patients.^1^ One theory proposes that increased pressure in venous anomalies contributes to the development of dilated ectatic microvasculature, presenting as telangiectasia. This may explain the close anatomic location of these anomalies.^14^ Another theory suggests that venous vascular malformations are fragile enough to cause microhaemorrhages which can trigger reactive angiogenesis.^16^

MRI with gadolinium contrast is the diagnostic modality of choice, which can differentiate BCT from other vascular anomalies, particularly cavernous angiomas, which can be diagnostically similar.^17^ BCT appears as isointense lesions with elevated edges.^17^ While T1 and T2 images may appear normal, images taken post‐contrast in T1 may show a capitation in a pointed form or “brush” pattern that is characteristic of BCT. It can appear as an isolated lesion with a dotted appearance or as multiple dotted foci. These findings are even more highlighted in the susceptibility‐weighted imaging (SWI) study, which provides a precise submillimetre definition.^16^, ^17^

Conservative management is typically preferred in most cases of BCT given its benign nature and extremely low risk of bleeding and disease progression.^5^, ^17^ However, in minority of patients, both surgical intervention and endovascular treatment are recommended and equally appropriate approaches.^17^

In conclusion, BCT can be asymptomatic and manifest with variable neurological symptoms as well. While the exact etiology of BCT remains uncertain, it is typically a benign small vascular malformation and may be associated with venous anomalies. MRI is the preferred diagnostic modality, and conservative management is usually recommended, although surgical removal and endovascular treatment are options for symptomatic cases.

## AUTHOR CONTRIBUTIONS

**Moaz O. Moursi:** Writing – original draft; writing – review and editing. **Anas Alsadi:** Writing – original draft; writing – review and editing. **Jouhar Kolleri:** Writing – original draft. **Yousra Ali:** Writing – original draft. **Tanweer Hussein:** Conceptualization; supervision; writing – review and editing.

## FUNDING INFORMATION

Open Access funding provided by the Qatar National Library.

## CONFLICT OF INTEREST STATEMENT

There is no conflict interest to be declared.

## ETHICS STATEMENT

All methods were performed in accordance with the relevant guidelines and regulations. Written informed consent was obtained from the patient.

## CONSENT

Written informed consent was obtained from the patient for publication of this case report and any accompanying images. A copy of the written consent is available for review by the Editor‐in‐Chief of this journal on request.

## Data Availability

Data sharing is not applicable to this article as no datasets were generated or analyzed during the current study.
